# Noteworthy Facts about a Methane-Producing Microbial Community Processing Acidic Effluent from Sugar Beet Molasses Fermentation

**DOI:** 10.1371/journal.pone.0128008

**Published:** 2015-05-22

**Authors:** Aleksandra Chojnacka, Paweł Szczęsny, Mieczysław K. Błaszczyk, Urszula Zielenkiewicz, Anna Detman, Agnieszka Salamon, Anna Sikora

**Affiliations:** 1 Institute of Biochemistry and Biophysics Polish Academy of Sciences, Warsaw, Poland; 2 Faculty of Biology, Warsaw University, Warsaw, Poland; 3 Faculty of Agriculture and Biology, Warsaw University of Life Sciences, Warsaw, Poland; 4 Institute of Agricultural and Food Biotechnology, Warsaw, Poland; Oak Ridge National Laboratory, UNITED STATES

## Abstract

Anaerobic digestion is a complex process involving hydrolysis, acidogenesis, acetogenesis and methanogenesis. The separation of the hydrogen-yielding (dark fermentation) and methane-yielding steps under controlled conditions permits the production of hydrogen and methane from biomass. The characterization of microbial communities developed in bioreactors is crucial for the understanding and optimization of fermentation processes. Previously we developed an effective system for hydrogen production based on long-term continuous microbial cultures grown on sugar beet molasses. Here, the acidic effluent from molasses fermentation was used as the substrate for methanogenesis in an upflow anaerobic sludge blanket bioreactor. This study focused on the molecular analysis of the methane-yielding community processing the non-gaseous products of molasses fermentation. The substrate for methanogenesis produces conditions that favor the hydrogenotrophic pathway of methane synthesis. Methane production results from syntrophic metabolism whose key process is hydrogen transfer between bacteria and methanogenic *Archaea*. High-throughput 454 pyrosequencing of total DNA isolated from the methanogenic microbial community and bioinformatic sequence analysis revealed that the domain *Bacteria* was dominated by *Firmicutes* (mainly *Clostridia*), *Bacteroidetes*, *δ*- and *γ-Proteobacteria*, *Cloacimonetes* and *Spirochaetes*. In the domain *Archaea*, the order *Methanomicrobiales* was predominant, with *Methanoculleus* as the most abundant genus. The second and third most abundant members of the Archaeal community were representatives of the *Methanomassiliicoccales* and the *Methanosarcinales*. Analysis of the methanogenic sludge by scanning electron microscopy with Energy Dispersive X-ray Spectroscopy and X-ray diffraction showed that it was composed of small highly heterogeneous mineral-rich granules. Mineral components of methanogenic granules probably modulate syntrophic metabolism and methanogenic pathways. A rough functional analysis from shotgun data of the metagenome demonstrated that our knowledge of methanogenesis is poor and/or the enzymes responsible for methane production are highly effective, since despite reasonably good sequencing coverage, the details of the functional potential of the microbial community appeared to be incomplete.

## Introduction

There is currently great interest in the development of new technologies for the production of energy from renewable sources, of which fermentation processes generating methane and hydrogen show great promise. Methane and carbon dioxide are the main final products of the decomposition of biomass under anaerobic conditions in environments where the concentration of other electron acceptors, such as nitrate, sulfate, Fe(III) and Mn(IV), is low. Such anaerobic digestion is a complex process that requires the interaction of many groups of microorganisms responsible for, respectively, hydrolysis, acidogenesis (mainly hydrogen-yielding fermentations), acetogenesis (mainly syntrophic degradation of fermentation products) and methanogenesis [1]. For the purposes of biotechnology it is desirable to separate the hydrogen- (hydrolysis and acidogenesis) and methane-yielding (acetogenesis and methanogenesis) stages to respectively favor the production of hydrogen and methane under controlled conditions. In the first dark fermentation stage, hydrogen-rich fermentation gas is produced, while in the second stage, the non-gaseous products of hydrogen fermentation act as substrates for methanogenic communities. These two processes are carried out in separate bioreactors with different pH conditions and hydraulic retention times (HRTs) [2].

The known cultured methanogens are strict anaerobes and comprise seven orders in the class *Euryarchaeota* of the *Archaea* domain: *Methanobacteriales*, *Methanococcales*, *Methanomicrobiales*, *Methanosarcinales*, *Methanopyrales* [3, 4], *Methanocellales* [5] and *Methanomassiliicoccales* [6]. Methane production from three groups of substrates proceeds via three methanogenic pathways: (i) splitting of acetate (aceticlastic or acetotrophic methanogenesis); (ii) reduction of CO_2_ with H_2_ or formate and rarely ethanol or secondary alcohols as electron donors (hydrogenotrophic methanogenesis); and (iii) reduction of methyl groups of methylated compounds such as methanol, methylated amines or methylated sulfides (methylotrophic methanogenesis). Surprisingly only two known genera, *Methanosarcina* and *Methanosaeta* (members of the order *Methanosarcinales*) are capable of methane production from acetate. Most of known methanogens produce methane by the reduction of CO_2_ [3–5, 7–9]. The known members of the *Methanomassiliicoccales* are H_2_-dependent methylotrophs [6]. The dominant type of methanogenesis is determined by the environmental/reactor conditions and it is thought that two-thirds of the methane generated in anaerobic digesters is produced from acetate [1, 4].

Due to the limited number of substrates for methanogenesis, methanogens are strictly dependent on partner microbes with which they form syntrophic relationships. The partner microbes oxidize fermentation intermediates (e.g. butyrate or propionate) to acetate, formate, carbon dioxide and hydrogen that are directly used by methanogens, thus making the syntrophic system efficient and thermodynamically favorable. The basis of this syntrophic cooperation is reverse electron and interspecies hydrogen transfer [8–12].

Since the required pH for methane generation in bioreactors is between 6.8–8.5 and any decrease disturbs the methanogenic process, controlled two-stage systems must provide stable conditions for the syntrophic transformation of non-gaseous products of hydrogen-yielding fermentations into methane. A growing number of reports describe the use of two-stage systems for hydrogen and methane production at the laboratory and pilot scales using various substrates [2, 13–18]. The idea of two-phase anaerobic digestion as a method for the effective degradation of biomass to methane and carbon dioxide is not new [19]. However, efficient methane production from non-gaseous fermentation products could make biological production of hydrogen via fermentation economically viable [2]. So far, studies on the co-production of hydrogen and methane by the anaerobic digestion of biomass have focused on the performance and efficiency of the entire process, but they have lacked any in-depth analysis of the microbial communities in the bioreactors where the two steps are performed. A good understanding of the structure and diversity of hydrogen- and methane-generating microbial communities, capable of syntrophic cooperation in the transformation of substrate to the desired gaseous products, should facilitate the optimization of hydrogen and methane co-production from organic matter in two-stage systems.

There have been numerous reports describing metagenomic analyses of methane-producing microbial communities present in bioreactors using different substrates as a feedstock. The examined samples came from full-scale biogas reactors [20–23] or laboratory-scale bioreactors [24–26]. Rademacher and co-workers [27] analyzed two microbial communities from a two-phase system fed with rye silage and winter barley straw under thermophilic conditions. However, they focused on carbohydrate degradation and not hydrogen production in the first stage.

Previously, we described an effective system for hydrogen production from sugar beet molasses and performed 454-pyrosequencing-based metagenomic analysis of the microbial community responsible [28]. Here we focus on the utilization of acidic effluent from molasses fermentation as a substrate for methane production in an upflow anaerobic sludge blanket (UASB) reactor. We have performed molecular analysis of the methane-yielding microbial community selected in the UASB bioreactor and studied the roles of specific groups of microorganisms in the processing of acidic effluent from molasses fermentation into methane and carbon dioxide. Thus, the results of this study together with our earlier findings [28] provide a detailed molecular characterization of a two-stage anaerobic digestion system producing hydrogen (in stage 1) and methane (in stage 2) from sugar beet molasses as the primary energy substrate under mesophilic conditions.

## Materials and Methods

### Seed sludge, feed composition and experimental set-up for continuous methane production

The object of this study was to obtain and characterize a methane-yielding microbial community processing acidic effluent from molasses fermentation that constituted stage 2 of a continuous two-step anaerobic digestion system aimed at hydrogen and methane production. Stage 1 of this system, the source of acidic effluent, was described previously [28]. The seed methanogenic inoculum was activated sludge from a municipal waste treatment plant “Warszawa Poludnie” in Warsaw, Poland, sampled in the autumn. The director of Municipal Water and Sewage Enterprise in the capital city of Warsaw in Poland issued the permission to sample activated sludge and use it for scientific research. A 3.5-litre UASB reactor was filled with the activated sludge (1.5 L) and neutralized effluent from molasses fermentation (2 L) and incubated at room temperature (20–25°C) for 5 days. Neutralization of the effluent with calcium hydroxide (50 g/L) was performed in a separate tank. After this time, gas production was observed and neutralized fermentation effluent was then continuously supplied to the UASB reactor using a peristaltic pump (ZALIMP, Poland), with an HRT of 7 days. A stable bilayer structure formed after 25 days of cultivation, consisting of methanogenic sludge and a liquid phase that occupied 75 and 25% of the bioreactor, respectively. To avoid decreasing the pH in the UASB bioreactor, neutralization of the acidic effluent from molasses fermentation was required for the first 68 days of cultivation. After this time, non-neutralized acidic effluent was supplied directly to the bioreactor.

### Analytical methods

The pH of the acidic effluent from molasses fermentation and the methanogenic effluent was measured using a standard pH meter (ELMETRON model CP-502). The chemical oxygen demand (COD) of all liquids was determined using a NANOCOLOR COD 1500 kit (Machery-Nagel) according to ISO 1575:2002. The COD of both centrifuged (bacterial cells removed) and non-centrifuged samples of the effluents was determined.

The total rate of gas production was measured using a MilliGascounter MGC-1 (RITTER) or a 270-cm^3^ gas pipette (filled with saturated KCl solution and connected to the bioreactor via a gas-tight junction). The composition of fermentation gas was analyzed by GC/TCD (gas chromatography with thermal conductivity detector) and GC/FPD (gas chromatography with flame photometric detector) according to ISO 19739, using an Agilent Technologies model 7890A gas chromatograph.

Samples of the acidic effluent from molasses fermentation and the methanogenic effluent were centrifuged twice and the concentrations of carbohydrates (sucrose, glucose and fructose), short-chain fatty acids and ethanol in these supernatants were determined. The carbohydrates were analyzed using high performance liquid chromatography (HPLC) with refractometric detection (Waters HPLC system: Waters 2695—Separations Module, Waters 2414—Refractive Index Detector, and 300×6.5 mm Sugar Pak column with guard column). Short-chain fatty acids were analyzed by HPLC with photometric detection (Waters HPLC system as above, Waters 2996—Photodiode Array Detector, and 300×7.8 mm Aminex HPX-87 H column with guard column). Ethanol was quantified by gas chromatography with flame-ionization detection (Hewlett Packard 6890, autosampler headspace—Hewlett Packard 7694E, polar 1.0-μm capillary column and FID). The HPLC conditions used for evaluating the levels of carbohydrates and organic acids were as described previously [28].

The Bradford assay was used to determine the concentration of proteins in the acidic effluent from molasses fermentation and the methanogenic effluent. Effluent samples were centrifuged, the supernatants were mixed with 2× concentrated extraction buffer (50 mM HEPES, 200 mM KCl, 24 mM MgCl_2_, 0.2 mM EDTA, 40% glycerol), while the pellets were resuspended in 1× extraction buffer (25 mM HEPES, 100 mM KCl, 12 mM MgCl_2_, 0.1 mM EDTA, 20% glycerol) and homogenized by vortexing with glass beads (Ø 0.40–0.60 mm, Sartorius). In addition, non-centrifuged effluent samples were also mixed with 2× concentrated extraction buffer and vortexed. All the samples were then centrifuged and the protein concentration determined in the supernatants using the Quick Start Bradford Protein Assay (Bio-Rad).

The concentration of sulfate in the acidic effluent from molasses fermentation was determined using a NANOCOLOR SULFAT 200 kit (Machery-Nagel). The content of Fe(II) in the effluent from the UASB bioreactor was determined as described previously [29].

The pH and COD values, the concentration of carbohydrates, short-chain fatty acids, ethanol, proteins, sulfate and Fe(II) in the examined effluents, the total rate of gas production and its composition presented in this study come from analyses performed on samples collected from the UASB reactor between the 13^th^ and 15^th^ week of the process. In each case a mean ± SD (standard deviation) were calculated.

### Total DNA isolation and pyrosequencing

Total DNA from the methanogenic community formed in the UASB reactor was extracted from a representative sample taken in the 14^th^ week of the process according to the following procedure. Samples of methanogenic sludge were collected from the reactor using a sterile spoon and 15 ml aliquots were centrifuged at 22,000 g for 5 min. The pelleted material was resuspended in extraction buffer (1.5 mM NaCl, 100 mM Tris-HCl, 100 mM sodium EDTA, 100 mM sodium phosphate; pH 8.0) and homogenized by vortexing for 15 s with glass beads (Ø 0.40–0.60 mm, Sartorius). Each sample was then incubated with proteinase K (5 mg/ml) and lysozyme (30 mg/ml) at 37°C for 1 h and subsequently with 1% SDS at 65°C for 10 min. Following the addition of more extraction buffer, the sample was centrifuged at 4600 g for 10 min. The supernatant was then extracted with chloroform-isoamyl alcohol (24:1) and centrifuged at 22,000 g for 3 min. This extraction was repeated several times to remove all proteins. The isolated genomic DNA was precipitated from the final aqueous phase with isopropanol and the pellet washed with 70% ethanol before resuspending in molecular grade H_2_O. The purified genomic DNA samples were sequenced using a shotgun approach with a GS FLX Titanium (454) pyrosequencer. Shotgun library construction was performed using approximately 5 μg of genomic DNA following the manufacturer's instructions with slight modification: DNA was fragmented by nebulization at 13 psi (0.87 bar), purified on a spin column (Qiagen), separated by electrophoresis on a 1% agarose gel, and fragments in the range 500–1100 bp were isolated by gel extraction (Qiagen). Library samples were sequenced on one large region of a PicoTiterPlate (1/2 PTP). Two independent sequencing runs were performed (smaller and larger), yielding over 600,000 reads in total. All raw sequences generated in this study have been deposited in the NCBI short reads archive under accession number SRR1611798.

### Sequence analysis

All sequence reads were used for similarity searches against the NR database, employing the program RAPsearch2 [30] with an E value cutoff of 0.001. Based on the results of these searches, taxonomy assignment was made using MEGAN5 [31] using default parameters (minScore = 50.0, maxExpected = '0.01', topPercent = 10.0, minSupport = 50, minComplexity = 0.44). Analysis of the abundance of microbial pathways was performed with HUMAnN software [32] on the basis of the results of another RAPSearch2 run with all reads, using an Evalue of 0.001 as the threshold. The reference database consisted of proteomes of the major Archaeal species identified by MEGAN5 (if available) and selected high quality proteomes obtained from the KEGG (Kyoto Encyclopedia of Genes and Genomes) database [33] as suggested by the HUMAnN developers. Metagenome assembly was performed with MetaVelvet version 1.1 using a maximal kmer length of 31 and automated peak detection [34]. Assembled contigs were mapped to bacterial and archaeal genomes from the NCBI database using the BWA MEM algorithm [35].

### Microscopic observations and elemental analysis

Samples of fresh methanogenic sludge collected from the UASB bioreactor between the 13^th^ and 15^th^ week of the process were spread out on a glass Petri dish, dried in air at room temperature and examined with a scanning electron microscope (JEOL, JSM-6380LA, Analytical Scanning Electron Microscope) using the following conditions: LW vacuum 40 Pa, accelerating voltage 20 kV, working distance 10 mm. The microscope was equipped with an EDS (Energy Dispersive X-ray Spectroscopy) analyzer (life time 100 s) to enable qualitative elemental analysis of the examined specimens. In addition, air-dried samples of the methanogenic sludge were subjected to X-ray diffractometry with an X’Pert-PRO MPD X-ray diffractometer system (PANalytical), using the DSH method with Co radiation. The methanogenic sludge was also Gram-stained and examined with a light microscope (Nikon Eclipse E200; 100× objective lens).

## Results and Discussion

### Biodiversity of the microbial community processing acidic effluent from molasses fermentation—general overview

Knowledge on the biodiversity and functioning of microbial communities processing non-gaseous products of dark fermentation to methane is still limited. Therefore, we have established a two-stage anaerobic digestion system producing hydrogen (in stage 1) and methane (in stage 2) from sugar beet molasses as the primary energy substrate under mesophilic conditions in a long-term continuous system. Stage 1 was described previously [28]. The focus of the present study was the analysis of the methane-yielding microbial community, based on syntrophic relationships between methanogenic *Archaea* and acetogenic *Bacteria*, processing the acidic effluent from molasses fermentation in a UASB reactor. Such bioreactors are usually employed to develop methanogenic microbial communities and to promote the formation of granules that show high methanogenic activity [36, 37].

**Fig 1:**
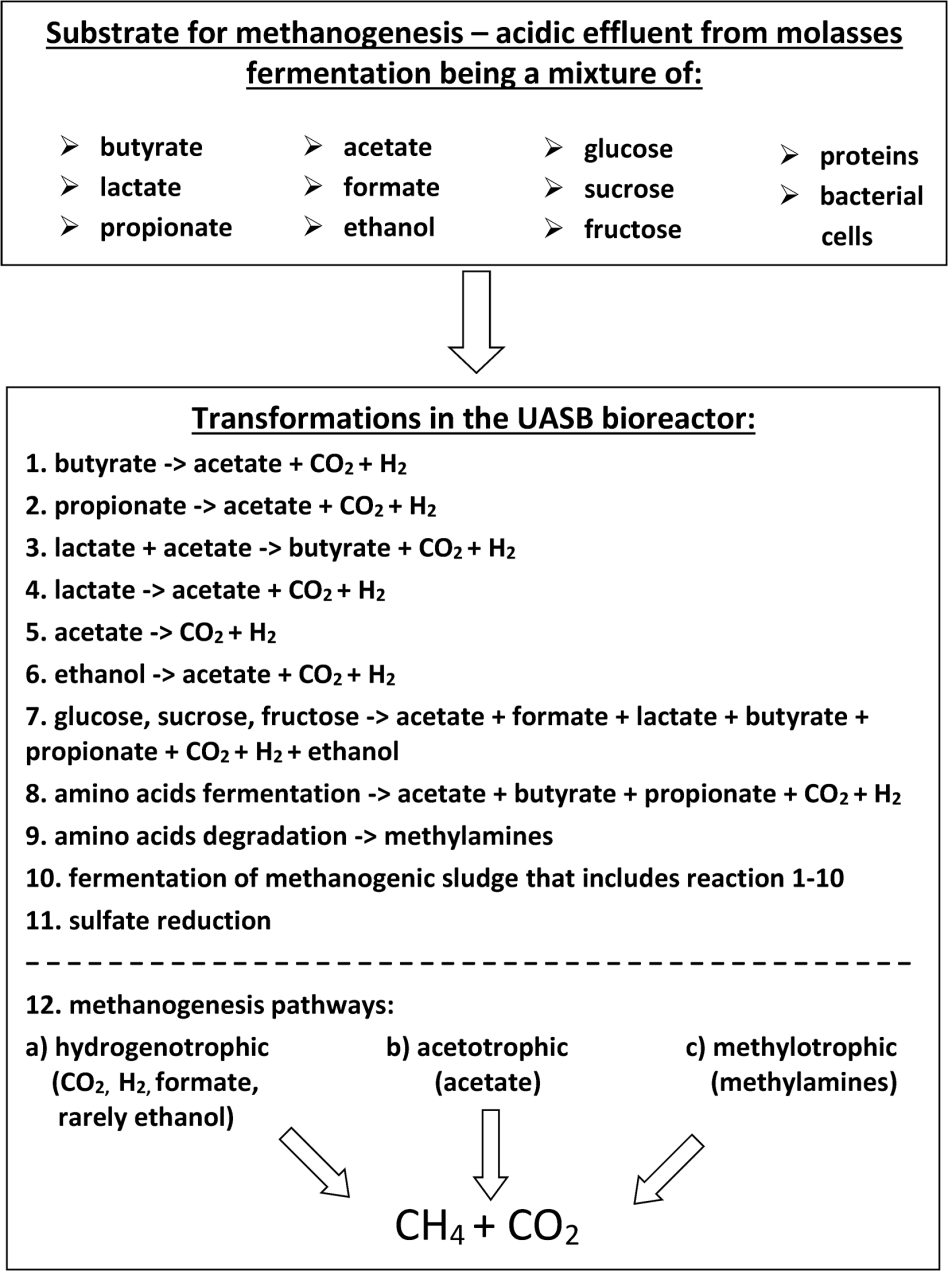
Rarefaction curve obtained from MEGAN5 classification. The curve does not flatten within our sequencing depth, but the increase in the number of leaves in taxonomy is small (ca. 150 leaves with minimal support of 50 per 100,000 reads).

**Fig 2:**
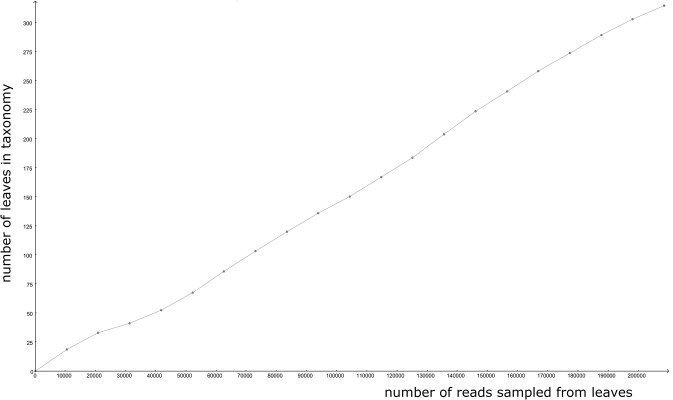
Methane-yielding community composition based on taxonomic assignments from 454-pyrosequencing reads generated using MEGAN5: (A) total reads; (B) reads assigned to the *Bacteria* domain, (C) reads assigned to the *Archaea* domain.

The microbial community fed with the acidic effluent from molasses fermentation formed tiny, loose granules occupying 75% of the UASB bioreactor. Total DNA was isolated from the community and sequenced using 454-pyrosequencing. Over 630,000 reads were obtained in two independent sequencing runs. Of these, around 500,000 reads were checked against the NR database (the rest were low complexity artifacts) and approximately 400,000 gave at least one significant hit. Taxonomic assignment revealed a broad but relatively simple distribution of microorganisms, with several genera being responsible for methanogenesis. A rarefaction curve calculated with MEGAN5 (Fig 1) indicates the presence of a long tail of microbial diversity, since the curve has no tendency to flatten. To check the consistency of taxonomic assignment from the sequence reads, we independently assembled the metagenome of the largest of the two runs, consisting of 496,000 reads, and used Rapsearch2 to perform a search against the NR database. The results of taxonomic assignment of the assembled metagenome with MEGAN5 showed a large degree of similarity to the picture obtained from the reads, confirming the structure of the biodiversity in the sample (Fig 2 and S1 Table). In summary, of 516,638 reads, 403,816 could be assigned to cellular organisms, 91 to viruses and the rest constituted low-complexity (80,062), non-assigned (30,035) or unclassified (97) sequences. For 2547 reads, MEGAN5 could not differentiate between cellular organisms and viruses. Among cellular organisms, 308,946 reads were assigned to *Bacteria*, 77,818 to *Archaea* and 355 to *Eukaryota*. Thus the apparent ratio of *Bacteria* to *Archaea* in the methanogenic community was 4:1. Contigs obtained during assembly were mapped onto bacterial and archaeal genomes from the NCBI database. Only a few genomes were significantly (> 30%) covered by contigs: *Methanoculleus marisnigri* JR1, *Methanoculleus bourgensis* MS2T, *Methanosaeta concilii* GP6 and *Methanocorpusculum labreanum* Z. In addition, we recovered significant portions of two plasmids: p5482 of *Bacteroides thetaiotaomicron* VPI-5482 and pBACSA03 of *Bacteroides salanitronis* DSM 18170.

### Performance of the microbial community processing acidic effluent from molasses fermentation

The two-stage continuous system producing hydrogen and methane was monitored regularly. The data in Tables 1 and 2 describe the representative performance of the studied methane-yielding microbial community at HRT = 7 days, when samples were taken for metagenomic analysis. As shown in Table 2, methane was produced at the rate of 2.12 L/L-reactor/d which corresponds to 0.46 L/g COD of non-centrifuged acidic effluent from molasses fermentation. Table 1 summarizes the characteristics of the acidic effluent resulting from molasses fermentation in stage 1 that acts as the substrate for methanogenesis, and the effluent from the methanogenic process itself (stage 2).

**Table 1:** Characteristics of the acidic effluent resulting from molasses fermentation used as the substrate for methanogenesis, and the effluent from the methanogenic process.

Parameter	Substrate for methanogenesis (acidic effluent from molasses fermentation)		Effluent from methanogenic process	
COD (g O_2_ /L)				
centrifuged	29.6 ± 0.7	n = 10	5.1 ± 0.1	n = 4
non-centrifuged ^1^	32.4 ± 4.0	n = 4	8.4 ± 0.6	n = 4
concentration of:				
sucrose (mg/L)	600 ± 500	n = 3	< 0.01	n = 3
glucose (mg/L)	80 ± 80	n = 3	< 0.01	n = 3
fructose (mg/L)	450 ± 130	n = 3	< 0.01	n = 3
formic acid (mg/L)	8 ± 0.9	n = 3	31.0 ± 7.0	n = 3
acetic acid (mg/L)	1061 ± 33.4	n = 3	1306 ± 13.8	n = 3
butyric acid (mg/L)	7959 ± 912	n = 3	272 ± 30.0	n = 3
isobutyric acid (mg/L)	438 ± 91.2	n = 3	83.0 ± 6.0	n = 3
lactic acid (mg/L)	1351 ± 23.7	n = 3	< 1	n = 3
propionic acid (mg/L)	561 ± 72.4	n = 3	912 ± 12.5	n = 3
ethanol (mg/L)	600 ± 50	n = 3	3.0	n = 3
proteins (mg/L) ^2^	50.21 ± 6.5	n = 4	104.5 ± 18.1	n = 4
sulfate (mg/L)	110.2 ± 6.6	n = 5	100.5 ± 7.0	n = 4
Fe(II) (mM)	< 0.1	n = 5	< 0.1^3^	n = 3
pH	5.00 ± 0.03	n = 12	7.21 ± 0.21	n = 12

^1^ not-centrifuged effluents contained microbial cells

^2^ the protein concentrations were determined in non-centrifuged (containing microbial cells) samples, while the concentrations of the other components were determined in centrifuged samples.

^3^ the content of Fe(II) in the methane-yielding granular sludge in the UASB bioreactor was 5.2 ± 0.7 mM (n = 3).

**Table 2:** Characteristics of biogas generated from the acidic effluent of sugar beet molasses fermentation by the methanogenic microbial community in the UASB bioreactor.

**Total biogas production:**		
L/working volume of the bioreactor/d	12.7 ± 0.39	n = 10
L/L-reactor/d	3.63 ± 0.11	
**composition of biogas** (%):		
methane	59.4 ± 1.3	n = 3
carbon dioxide	37.9 ± 1.0	n = 3
water vapor	1.4 ± 0.3	n = 3
hydrogen	0.3 ± 0.3	n = 3
H_2_S (ppm)	156.4 ± 20.0	n = 3
**methane production**:		
L-CH_4_/working volume of the bioreactor/d	7.43 ± 0.85	
L/L-reactor/d	2.12 ± 0.24	

The total COD of the substrate for methanogenesis was very high. This confirmed the presence of a high concentration of non-gaseous end products typical for hydrogen-yielding dark fermentation. The total COD of the effluent from the UASB bioreactor was decreased 4–6 times depending on whether centrifuged or non-centrifuged effluents were compared. This indicates the efficient utilization of components of the acidic effluent by the methane-yielding microbial community.

The acidic effluent from molasses fermentation contained sucrose, glucose and fructose, sugars that had not been fermented in stage 1, but which were completely utilized in the methanogenic step (stage 2). The analysis of short-chain fatty acids revealed that butyric acid was the main substrate used by the methanogenic community. Complete utilization of lactic acid by the methane-producing community was also observed. Acetic acid and traces of formic acid detected in the effluent from the UASB bioreactor probably came from the oxidation of butyric and lactic acids. The same was observed by Park and colleagues [15]. Interestingly, the concentration of propionic acid in the effluent from stage 2 was high. Since the effluent from stage 1 contains proteins, propionic acid was probably produced by syntrophic oxidation of amino acids, and its subsequent utilization in stage 2 was somehow inhibited. The presence of high concentrations of propionate in this type of system has been reported previously [38]. The microbes involved in the metabolism of propionate will be discussed below.

**Fig 3:**
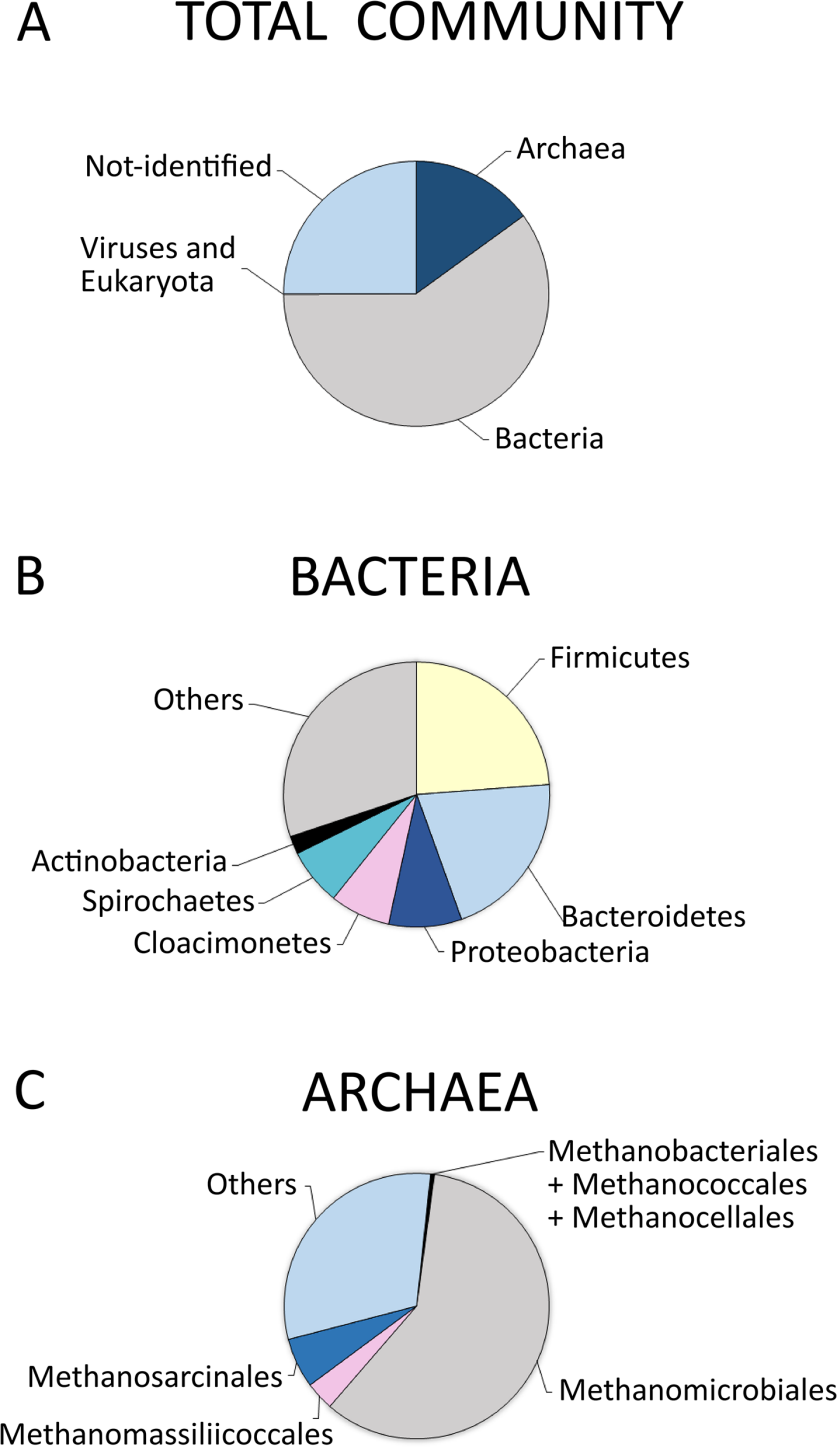
The expected metabolic pathways used for transformation of the components of the acidic effluent from sugar beet molasses fermentation to methane and carbon dioxide in the UASB bioreactor.

The expected metabolic pathways utilized in the transformation of the acidic effluent components to methane and carbon dioxide in the UASB bioreactor are presented in Fig 3. Phylogenetic characterization of the methane-producing microbial community from total DNA sequence data of the granular sludge formed in the UASB bioreactor revealed the dominant and subdominant groups of microorganisms. The assignment of metabolic pathways leading to methane formation to specific taxa is discussed below.

### Taxonomic distribution and role of *Bacteria* in the methanogenic community

Detailed analysis of the DNA sequence reads permitted description of the taxonomic distribution of bacteria in the methane-yielding microbial community in the UASB bioreactor. The percentage values quoted below refer to the proportion of bacterial reads. The domain *Bacteria* was dominated by *Firmicutes* (~ 24%), *Bacteroidetes* (~ 21%), *Proteobacteria* (~ 9%), *Cloacimonetes* (~ 7.5%) and *Spirochaetes* (~ 7%). The predominant *Firmicutes* were *Clostridia*, which constituted approximately 14% of all bacterial reads. The *Proteobacteria* were mostly represented by the delta and gamma subdivisions (~ 9% and ~ 1.5%, respectively), whereas the alpha and beta subdivisions were poorly represented (~ 0.5%). Other minor groups were *Actinobacteria* (~ 2%), *Chlamydiae* (~ 1%), *Synergisetes* (~ 1%) and *Chloroflexi* (~ 0.5%). A small number of reads were sequences from *Armatimonadetes*, *Negativicutes* and *Plantomycetes*.

The *Clostridia* and *Bacteroidetes* are abundant groups of bacteria in most methane-yielding bioreactors described in the literature. Since the majority of these studies examined one-step anaerobic digesters, these bacteria are thought to be responsible for the first steps in anaerobic digestion, i.e. the hydrolysis and dark fermentation as well as acetogenesis (secondary fermentation) [24, 26]. However, the acidic effluent of molasses fermentation that formed the substrate for methanogenesis in this study contained a low concentration of unfermented sugars (~1.1 g/L) that could be utilized by bacteria in hydrolysis and hydrogen-yielding fermentation (Fig 3, pathway 7). The low amount of unfermented sugars and the abundance of *Clostridia* and *Bacteroidetes* indicate that in this case, these bacteria primarily play a role in acetogenesis involving syntrophic oxidation of non-gaseous products of molasses fermentation. However, the *Syntrophomonadaceae*, highly specialized syntrophic microbes that can oxidize butyric, propionic and long-chain fatty acids (Fig 3, pathways 1 and 2) to acetic and formic acids with the production of hydrogen and carbon dioxide [39–42], constituted only 1.4% of the studied microbial community. It was represented by two species, *Syntrophomonas wolfei* and *Syntrophothermus lipocalidus*. Well-recognized propionate-oxidizers of the *Desulfotomaculum* and *Pelotomaculum* genera from the *Peptococcaceae* family (*Clostridiales*) constituted only about 0.9%, and butyrate-oxidizing *Syntrophus acidotrophicus* and propionate-oxidizing *Syntrophobacter fumaroxidans* from the order *Syntrophobacterales* (*Deltaproteobacteria*) only about 1.5% of the bacterial reads. The efficient utilization of butyric acid by the methane-yielding community (Table 1), despite the apparently low number of butyrate-oxidizing bacteria identified here, indicates that most of the fatty-acid oxidizers have not yet been recognized.

While relatively little is known about syntrophic oxidation of lactate, the data presented in Table 2 and the study of Park and colleagues [15] show that lactate is completely utilized by methane-yielding microbial communities fed with acidic effluent from molasses fermentation. Many Clostridial species are able to convert lactate and acetate to butyrate and hydrogen. We have previously postulated that symbiotic interactions between lactic acid bacteria and clostridia, referred to as lactate cross-feeding, exist in hydrogen-producing bioreactors. These reactions include hydrogen and butyrate production from lactate and acetate by Clostridial species [28, 43]. Here we hypothesize that the same phenomenon exists in methanogenic communities, but this time the products of lactic acid transformation constitute substrates for methanogenesis (Fig 3, pathway 3). The notion of lactate transformation in methanogenic communities has not previously been discussed or studied. Furthermore, representatives of the *Deltaproteobacteria*, *Desulfovibrio* species (~ 2%) are known to be capable of syntrophic growth on lactate and ethanol with hydrogenotrophic methane-producing partners (Fig 3, pathways 4 and 6) when other electron acceptors such as sulfate are absent. Otherwise, sulfate reduction occurs (Fig 3, pathway 11) [10, 44]. Traces of hydrogen sulfide detected in the biogas (Table 2) and the presence of sulfate in the acidic effluent from molasses fermentation (Table 1) suggest that some sulfate reduction was occurring in the UASB bioreactor.

Ethanol is another component of the acidic effluent from molasses fermentation that is effectively utilized by the methane-yielding microbial community (Table 1 and [15]). Apart from *Desulfovibrio* species, other well-recognized syntrophic ethanol-oxidizing representatives of the *Deltaproteobacteria* found in the UASB bioreactor are *Geobacter* and *Pelobacter—*well known Fe(III) reducers. To be an energetically effective reaction, the oxidation of ethanol to carbon dioxide and hydrogen also requires strict cooperation with hydrogenotrophic methanogens [10, 12]. Notably, the granular sludge in the UASB bioreactor and the effluent from this bioreactor were poor in Fe(II) compounds (Table 1).

The methanogenic sludge and the acidic effluent from molasses fermentation contained amino acids and proteins (Table 1), which are also transformed to methane (Fig 3, pathway 8). The pathways of amino acid fermentation differ depending on the amino acid type [10]. Furthermore, glutamate, for example, may be fermented via five different pathways [45]. Amino acids are generally degraded to acetate, propionate, hydrogen and carbon dioxide, with the formation of butyrate and ammonia. Members of the *Clostridiales* (*Clostridiaceae*, *Eubacteriaceae*, *Peptococcaceae*, *Peptostreptococcaceae* families), *Fusobacteriales* and *Synergistetes* (*Aminobacterium colombiense*) present in the methanogenic community are capable of amino acid fermentation [10, 12]. Transformation of amino acids to methane is only possible in syntrophic association with hydrogenotrophic methanogens that scavenge hydrogen, otherwise methane production would be energetically impossible.

Members of the *Cloacimonetes* were the third dominant group of bacteria detected in the methane-yielding UASB bioreactor. The *Cloacimonetes*, including candidate division WWE1 (Waste Water of Evry 1), are a sub-dominant group of bacteria found in anaerobic mesophilic digesters and gut microflora. So far, all attempts to cultivate representative of the *Cloacimonetes* have failed, probably due to their need for obligatory symbiotic relationships with other microorganisms. However, using metagenomic sequence data and genomic assembly procedures, the genome of a representative bacterium *Candidatus* Cloacimonas acidaminovorans has been reconstructed [46]. The candidate division WWE1 bacteria are regarded as syntrophs capable of amino acid fermentation, propionate and butyrate oxidation as well as cellulose degradation [12, 47]. Interestingly, *Candidatus* C. acidaminovorans died out in an anaerobic digester fed with protein substrates, probably due to the loss of the symbiotic partners and/or the lack of substrates such as short-chain fatty acids [48].

Acetate, the final product of syntrophic oxidation of butyrate, lactate, propionate, ethanol or amino acids, can be degraded to carbon dioxide and hydrogen by other syntrophs, namely acetate-oxidizing bacteria (Fig 3, pathway 5). Since acetate oxidation is an energetically unfavorable reaction, close cooperation with hydrogenotrophic methanogens is required. Known acetate-oxidizing bacteria belong to the following groups: *Synergistetes*—genera *Synergistes* [49]; *Clostridia*—*Thermoacetogenium phaeum*, *Moorella*, *Clostridium ultunense* or *sporomusa*; and the *Deltaproteobacteria*—*Geobacter* [10]. Representatives of all these bacteria were found in the methanogenic community processing acidic effluent from molasses fermentation. Thus, acetate-oxidizing bacteria appear to compete with acetotrophic methanogens for substrate in methane producing bioreactors.

Zheng and co-workers [50] showed that Clostridial species are key microorganisms in anaerobic sludge fermentation. Since the inoculum for the methanogenic community examined in the present study was activated sludge from waste water treatment, it is possible that Clostridial species participated in fermentation of the methanogenic sludge. It is noteworthy that sequences assigned to viruses are also present in the sample of the methane-yielding microbial community and these may be responsible for microbial cell lysis (S1 Table). Therefore, the methanogenic sludge itself could have supplied additional substrates such as carbohydrates, proteins and lipids for methanogenesis (Fig 3, pathway 11).

*Actinobacteria*, *Chlamydiae*, *Spirochaetes*, *Chloroflexi* and *Plantomycetes* are often among the bacterial phyla detected in methane-producing anaerobic digesters and wastewater treatment plants. Their functional activities are not well recognized in methanogenic communities. Some constituted accompanying groups of bacteria present in the inoculum used in this study (an activated sludge from a municipal sewage treatment plant). *Actinobacteria* and *Chloroflexi* are thought to hydrolyze and ferment carbohydrates. Interestingly, the contribution of *Chloroflexi* and *Plantomycetes* to butyrate oxidation was identified in experiments performed with [^13^C]-labelled butyrate by Liu and co-workers [40].

The results of the present study show that our knowledge of syntrophic metabolism is poor and incomplete because the majority of syntrophic bacteria are able to grow only in co-culture with a partner microbe. Thus, they are difficult to isolate and grow in pure culture, e.g. the candidate division WWE1 (*Cloacimonetes*) with no cultured representative so far. In the methanogenic community described here the partner microbe was most frequently a hydrogenotrophic methanogen. We postulate that the abundant groups of bacteria (*Firmicutes*, *Bacteroidetes*, *Proteobacteria*, *Cloacimonetes*, *Spirochaetes*) are mostly responsible for syntrophic oxidation of non-gaseous products of molasses fermentation. In addition, there are probably many unrecognized syntrophic bacteria among the minor groups (*Actinobacteria*, *Chlamydiae*, *Synergisetes*, *Chloroflexi*, *Plantomycetes*). Evidence supporting this notion may be found in the results of Liu and co-workers [40], which suggest that *Chloroflexi* and *Planctomycetes* can syntrophically oxidize butyrate.

### Taxonomic distribution and role of *Archaea* in the methanogenic community

Analysis of DNA sequences derived from the methanogenic community formed in the UASB bioreactor revealed that the order *Methanomicrobiales* predominated among *Archaea*, constituting about 59% of all reads from members of this domain (Fig 2). The percentage values given in parentheses below show the contributions of individual taxa. The most abundant genus within this order was *Methanoculleus* (37%) represented by *M*. *marisnigri* and *M*. *bourgensis*, while the second and third most abundant were *Methanocorpusculum* (10.8%, *M*. *labreanum*) and *Methanofollis* (5%, *M*. *liminatans*), respectively. Other representatives of this order were the genus *Methanoplanus* (*M*. *limicola* and *M*. *petrolearius*), and the species *Methanoregula formicica*, *Methanosphaerula palustris* and *Methanospirillum hungatei*. Other identified hydrogenotrophic methanogens were representatives of the *Methanobacteriales* including the genera *Methanobacterium*, *Methanococcales* and *Methanocellales* (*Methanocella arvoryzae*). These results indicate that the hydrogenotrophic pathway of methane synthesis is dominant in the bioreactor. *Archaea* conducting the aceticlastic pathway of methane production included the *Methanosarcinales* (~ 3.5%): genera *Methanosaeta* (~ 2.4%) represented by *M*. *concilii* (~ 2%) and *M*. *harundinacea*; as well as the *Methanosarcina* (~ 0.6%, *M*. *acetivorans*, *M*. *barkeri*, *M*. *mazei*). It is noteworthy that many *Methanosarcinales* can also use H_2_ to reduce CO_2_ [51].

Metagenomic analysis revealed a relatively high contribution of sequences assigned to the genus *Methanomassiliicoccus* (~ 4%), including *Methanomassiliicoccus luminyensis*, *Candidatus* Methanomassiliicoccus intestinalis and *Candidatus* Methanomethylophilus alvus (~ 2%). These represent the seventh recently described order of methanogens—the *Methanomassiliicoccales*. Initially isolated from human feces, these *Archaea* have been shown to be widely distributed in the environment. They use methylated compounds (mono-, di-, tri-methylamine and dimethylsulfide) as substrates for methanogenesis and the methyl group is reduced by hydrogen. This is H_2_-dependent methylotrophy [6]. Methylamines are products of anaerobic digestion of proteins. It is noteworthy that genome sequences of organisms conducting this pathway of methylotrophy constituted more than 7% of total Archaeal reads in this study.

When biomass is transformed into methane under mesophilic conditions in anaerobic digesters and fresh waters, it is first fermented to acetate, carbon dioxide and hydrogen, and formate, as well as short-chain fatty acids. Since the theoretical maximum hydrogen yield during dark fermentation occurs with the conversion of ^1^/_3_ of the substrate to hydrogen and carbon dioxide, and ^2^/_3_ of the substrate to acetate, it has been estimated that ^2^/_3_ of methane originates from acetate and ^1^/_3_ from hydrogen, formate and carbon dioxide [4]. However, culture-independent analyses of methanogenic communities, based on cloning and sequencing of 16S rDNA and *mcrA* gene fragments or high throughput DNA sequencing technologies, have revealed that the contribution of methanogens performing the aceticlastic or hydrogenotrophic pathways in anaerobic digesters depends on the type of substrate and the process conditions. *Methanomicrobiales* represented by *M*. *marisnigri* often predominate in methanogenic communities isolated from biogas plants, indicating that methane is produced via the hydrogenotrophic pathway. These results do not support the thesis that methane is produced primarily from acetate [21–24, 26, 52–54]. Our data also corroborate these contradictory findings and one explanation for these observations may be found in the amount of energy generated by the different pathways of methane formation. The acetoclastic pathway provides only a small amount of energy available for growth: CH_3_COO^—^+ H^+^ → CO_2_ + CH_4_ (ΔG^o^´ = –36 kJ/mol), whereas 4-fold more energy is produced by the hydrogenotrophic pathway: 4H_2_ + CO2 → CH_4_ + H_2_O (ΔG^o^´ = –131 kJ/mol), 4HCOO^—^+ 4H^+^ → CH_4_ + 3CO_2_ + H_2_O (ΔG^o^´ = –144.5 kJ/mol) [7]. Thus, the hydrogenotrophic pathway is much more energetically effective and this may be one of the reasons for the dominance of the *Methanomicrobiales* order in the analyzed communities, including that characterized in the present study. Analysis of substrate preferences of the recognized methanogenic *Archaea* revealed that hydrogen and carbon dioxide, methyl compounds and acetate are utilized by 74.5%, 33% and 8.5% of the methanogens, respectively [9]. On the other hand, only two genera, *Methanosaeta* and *Methanosarcina*, are recognized as acetotrophic methanogens. In all methanogenic communities examined by high throughput DNA sequencing, the contribution of unidentified sequences is usually high—in the present study it is 22%. Since phylogenetic analyses are based on DNA sequences present in databases and the majority of the recognized genera of methanogens produce methane via the hydrogenotrophic pathway, it is possible that acetoclastic methanogens are hidden among the unrecognized sequences. Therefore, the apparent dominance of hydrogenotrophic orders of methanogens such as *Methanomicrobiales* may be due to our limited knowledge of methanogenic *Archaea*. Attempts are being made to reconstruct genomes using tree reconciliation methods (Szczesny et al., in preparation), which should move unidentified sequences down in the taxonomic tree and aid the recognition of novel methanogenic species.

However, in the light of the present study, there are four strong arguments in favor of the dominance of the hydrogenotrophic pathway of methane generation in the UASB bioreactor processing acidic effluent from molasses fermentation. First, syntrophic oxidation of non-gaseous products of molasses fermentation generated significant amounts of hydrogen, formate and carbon dioxide used directly by methanogens. Second, methane production in a two-stage system provides pH stability, optimal for hydrogenotrophic methane synthesis. Any decrease in pH is known to inhibit the development and activity of hydrogenotrophic methanogens [4]. Third, the aceticlastic pathway of methane production as well as propionate oxidation by syntrophic bacteria could be inhibited by high concentrations of some minerals in the granular sludge, as discussed below. A decrease in the representation of *Methanosaetaceae* was previously found to be accompanied by an increase in the propionate concentration in methane-producing communities [38, 39]. Fourth, the acetate-oxidizing bacteria present in the UASB reactor supply substrates for hydrogenotrophic methanogens and compete for substrate with acetotrophic methanogens (discussed above).

The presence of representatives of the *Methanomassiliicoccales* within the characterized microbial community indicates that hydrogen-dependent methylotrophy is the second methane-yielding pathways in the described system. It also supports the notion that the degradation of proteins contributes to methane formation occurring in the UASB reactor.

Among the identified archaeal sequences some were assigned to non-methanogens: *Halobacteriaceae—*aerobic heterotrophs able to grow anaerobically [55]; *Thermoplasmata—*facultative anaerobes capable of sulfur respiration [56]*; Thermococcales*—anaerobes able to utilize proteins and carbohydrates [57]; *Archaeoglobus—*another anaerobe and known sulfate-reducer capable of oxidizing lactate to carbon dioxide [58]. The origin of the inoculum can explain the presence of these *Archaea* in the community. These organisms may have a negative influence on methane production due to substrate competition or H_2_S generation.

### Microscopic and elemental characteristics of the granules formed by the methane-yielding microbial community

**Fig 4:**
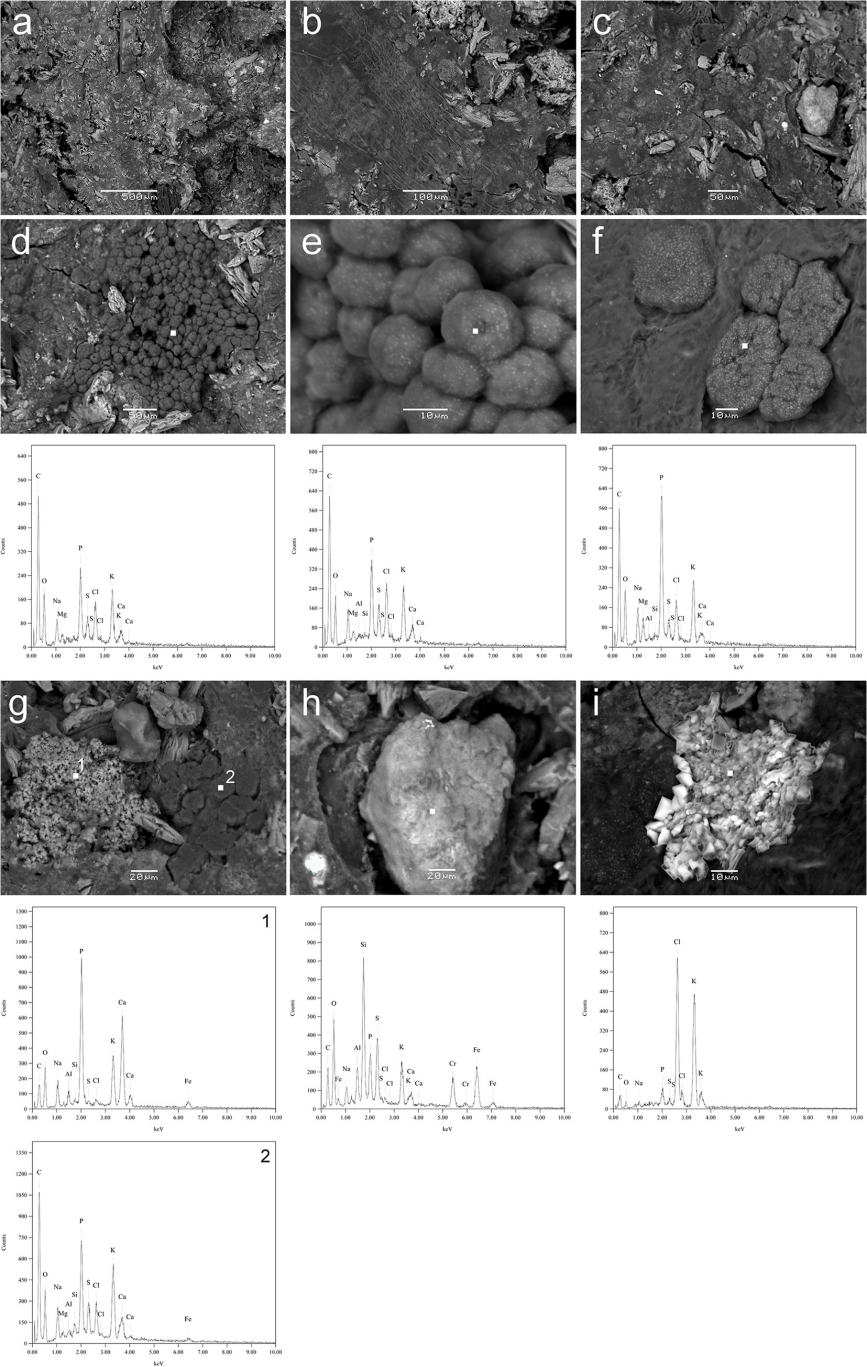
Scanning electron micrographs of heterogeneous methanogenic sludge from the UASB bioreactor: (a-c) general view; (d-e) granules, (g-i) matrix rich in minerals. Elemental analysis was performed by the generation of EDS spectra at the indicated points (squares).

Gram staining revealed that the granular structure formed by the methane-yielding microbial community in the UASB bioreactor is a complex of morphologically varied microbial cells surrounded by a matrix of extracellular material. Scanning electron microscopy showed that the matrix is highly heterogeneous and rich in minerals (Fig 4). At representative points in the scanning electron micrographs (indicated by squares in Fig 4), elemental analysis was performed to produce EDS spectra. This revealed the presence of the elements C, O, P, K, Ca, Al, Na, Cl, Mg, Fe and Si. X-ray diffraction analysis of methanogenic sludge samples identified the major inorganic components as the phosphates CaHPO_4_, AlPO_4_ and MgNH_4_PO_4_, as well as sylvite (KCl).

Methanogenic granules and effluents formed in the process of organic waste treatment are rich in minerals: mainly ferric sulfide and Ca-, Mg-, Na-, K- or Al-containing compounds. These originate from the breakdown of the biomass or the added chemicals, and constitute between 10 and 90% of the dry mass, depending on the composition of the wastes and nature of the methanogenic process [59]. Both Al and K are undesirable elements in the methanogenic sludge due to their competition with other essential metals, inhibition of microbial growth and consequently their adverse effect on the methanogenic process. In contrast, Ca and Mg have a positive effect due to their promotion of the granulation process. Sodium plays a role in the formation of ATP and oxidation of NADH, so is essential for the growth of methanogens. However, high concentrations of Ca^2+^, Mg^2+^ and Na^+^ ions cause inhibitory effects on methanogen activity. The optimum concentration of Ca^2+^ and Na^+^ ions for methane synthesis from acetate was found to be 200 mg/L and 230 mg/L, respectively, whereas a concentration of 8,000 mg/L of either ion inhibited the process [60]. Interestingly, the combination of various elements can mitigate the toxicity of the others, e.g. magnesium, sodium and ammonium counteract potassium toxicity. It is noteworthy that the aceticlastic pathway of methanogenesis and the oxidation of propionate are particularly sensitive to raised levels of certain minerals [60]. Moreover, it has been observed that inhibition of the acetotrophic pathway of methane formation is usually accompanied by inhibition of propionate oxidation [38]. The accumulation of minerals from the molasses and the M9 medium used in stage 1 (hydrogen production) may be one of the reasons for the dominance of hydrogenotrophic methane producers in stage 2 (Fig 2 and S1 Table). This might also explain high concentration of propionate in the effluents of the methane-producing bioreactor (Table 1). Similarly, Fang and co-workers [61] showed that sodium and potassium at concentrations of 11 and 28 g/L, respectively, inhibited methane production from desugared molasses. Taking into account data from the literature [61] and the composition of M9 medium [62], we estimate that the substrate for methanogenesis used in the present study contained (per liter) 63 mg, 2440 mg and 2250 mg of calcium, sodium and potassium, respectively. This suggests that the Ca^2+^ and Na^+^ ion concentrations were not optimal for methane synthesis from acetate.

We postulate that physical factors such as inorganic components of the extracellular matrix of methanogenic granules may inhibit some metabolic pathways and thus influence the processes leading to methane production by the microbial community. Thus, the substrate is a selecting factor determining the composition of the methanogenic community and the dominant pathway of methanogenesis.

### Functional analysis of the metagenome

**Fig 5:**
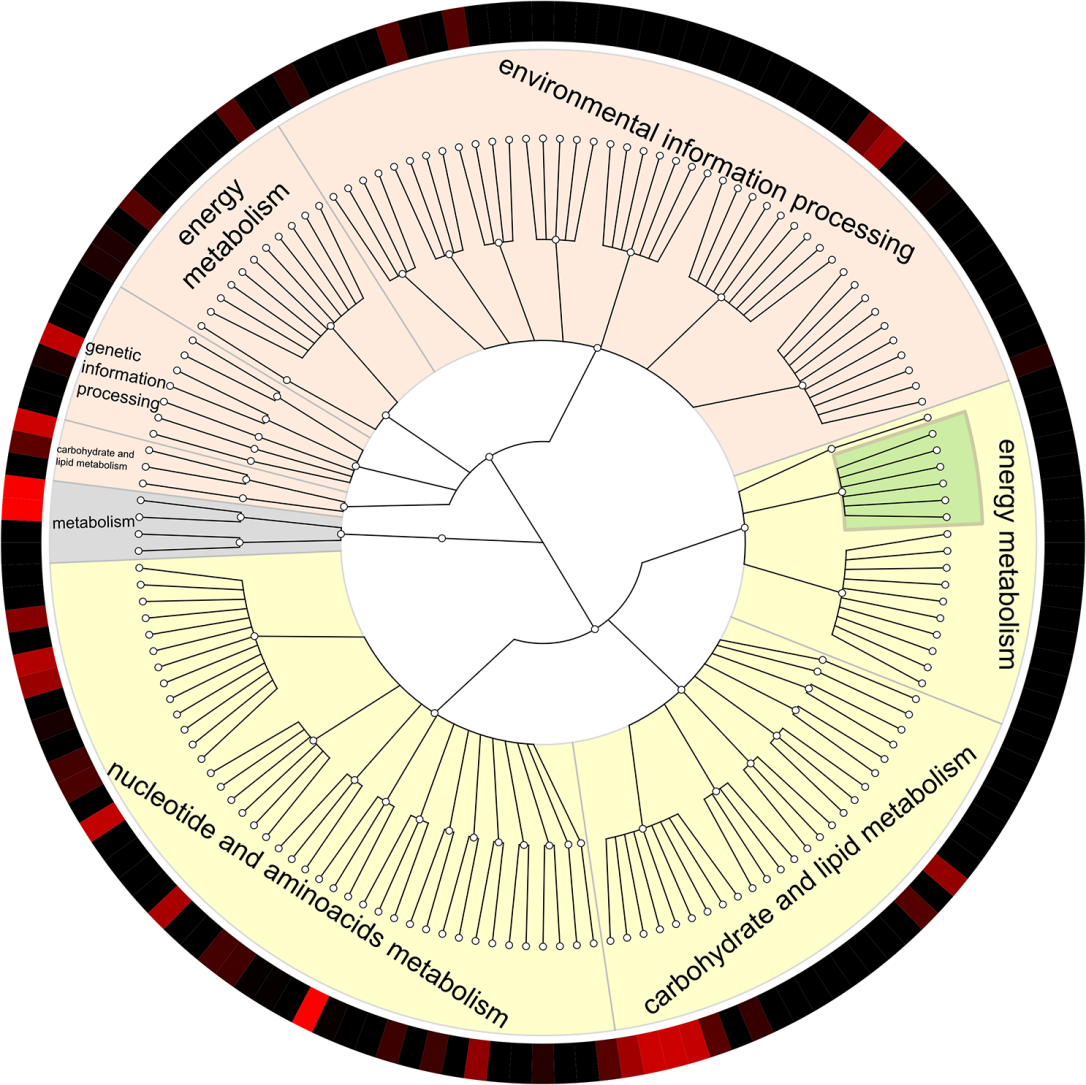
General overview of the functional capabilities of the microbial community in the UASB bioreactor processing acidic effluent from molasses fermentation, drawn with GraPhlAn software (http://huttenhower.sph.harvard.edu/graphlan). Yellow—pathway modules, light orange—structural complexes, grey—functional sets according to KEGG-based classification. Methane metabolism is highlighted in green. The color scale on the outermost ring represents low relative abundance (black) to high relative abundance (red).

The aforementioned roles of specific groups of microorganisms in the analyzed methanogenic community were verified by functional analysis of the metagenome to determine pathway abundance. The functional capabilities of the microbial community in the UASB bioreactor were determined by the identification of pathway modules, structural complexes and functional sets (Fig 5). This demonstrated that the most abundant pathway modules are from the following categories: (i) carbohydrate and lipid metabolism—central carbohydrate metabolism, i.e. glycolysis, pentose phosphate pathway, etc. and (ii) nucleotide and amino acid metabolism—biosynthesis of various amino acids. Pathway modules from the category of energy belong mainly to one of two groups: carbon fixation and methane metabolism. One module from sulfur metabolism was also identified. However, no module from the energy category stands out in terms of abundance. Unexpectedly, despite reasonably good sequencing coverage, the detailed functional potential of the community, as assessed with KEGG modules, appears incomplete. The biosynthesis of amino acids is present in high coverage [see S2 Table], but amino acid degradation modules seem to be largely lacking, except in the cases of methionine, leucine and histidine. The patchiness of the annotation is most visible in the case of the methane metabolic pathway, which is identified but does not have good coverage. Most of the modules in this pathway are missing, creating the impression that the assessed community is incapable of producing methane (see S1 Fig; selected enzymes from modules that HUMAnN assessed as significantly present are shown in pink). Some of the missing spots would probably be filled if the sequencing were deeper, but we believe that there is an alternative interpretation of this situation. Around 12.9% of all assigned reads were classified by sequence similarity to methanogenic species. Such low coverage of the methanogenesis process revealed during the analysis has to be related to the depth of reference annotations in functional annotation databases. The available analyses of amplicons of genes related to methanogenesis typically reveal an unexpected sequence diversity [63–65]. Either misclassification or a lack of annotation (many accessions are denoted “putative protein”), or low sequence similarity (100,000 reads lack similarity to known organisms) could be the reason for the patchy functional assignment. Thus, the results of our study emphasize the importance of careful interpretation of functional analyses from metagenomic surveys of methanogenic processes.

There is also the possibility that the low abundance of modules involved in methanogenesis reflects the true situation. The specific activity of methyl-coenzyme-M reductase (MCR) complex in *Methanothermobacter thermautotrophicus* is reported to be around 10–100 μmol/mg of protein/min [66, 67]. While the activity *in vivo* might be different, there is currently no evidence to indicate that the MCR complex is a rate limiting enzyme in methanogenesis. In the context of the bioreactor, which produces 0.3 moles of methane per day, the presumed activity of MCR corresponds to 4–40 μmoles of the complex, i.e. 1.2–12 grams of pure protein. Given that MCR is a highly abundant protein complex that can constitute up to 10% of total cell protein in *Methanobacterium thermoautotrophicum* [68], it is likely that even a small number of MCR-carrying microorganisms can supply enough of the enzyme for efficient methane production.

## Conclusions

Molecular characterization of a methane-producing microbial community processing acidic effluent from sugar beet molasses fermentation by analyzing the results of 454-pyrosequencing of total DNA revealed high biodiversity of the methanogenic sludge. Microorganisms of the hydrogenotrophic pathway of methane production are predominant in the UASB bioreactor and the most abundant methanogens are members of the *Methanomicrobiales*. *Firmicutes* (dominated by *Clostridia*), *Bacteroidetes*, *Proteobacteria* (dominated by delta- and gamma- subdivisions), *Cloacimonetes* and probably representatives of the *Chloroflexi* and *Plantomycetes* are responsible for syntrophic metabolism involving the transformation of short-chain fatty acids and proteins to substrates for methanogens. Since syntrophic metabolism in methanogenic communities involves interspecies hydrogen transfer, hydrogenotrophic methanogenesis is favored in the processing of acidic effluent from sugar beet molasses fermentation to methane. It is likely that mineral compounds present in the methanogenic sludge influence the functioning of the methanogenic community by inhibition/modulation of the syntrophic metabolism and methanogenic pathways. Functional analysis of the metagenome indicates that our knowledge of methanogenic communities and syntrophic metabolism in anaerobic digestion is still limited. On the other hand, the poor overlap between the phylogenetic analysis and functional analysis of the metagenome may reflect the high diversity of genes related to methanogenesis and/or the high efficiency of enzymes responsible for methane production.

## Supporting Information

S1 Fig Significant pathway modules (pink) in the methane metabolism map from the KEGG database. (TIF)

S1 Table Number of reads assigned to respective taxonomic branches by MEGAN5. (DOC)

S2 Table Abundance analysis of KEGG modules, classes and KEGG orthology groups obtained using HUMANn on the basis of sequence similarity to 28 genomes selected from the KEGG database. In addition, KO groups were mapped to EC numbers for selected enzymes putatively involved in methane metabolism (including intermediate and substrate production).
(XLS)
