# Supplementary figures and images for: Noteworthy Facts about a Methane-Producing Microbial Community Processing Acidic Effluent from Sugar Beet Molasses Fermentation
Source: PLoS One. 2015 May 22;10(5):e0128008. doi: 10.1371/journal.pone.0128008 (PMC4441513; doi:10.1371/journal.pone.0128008)

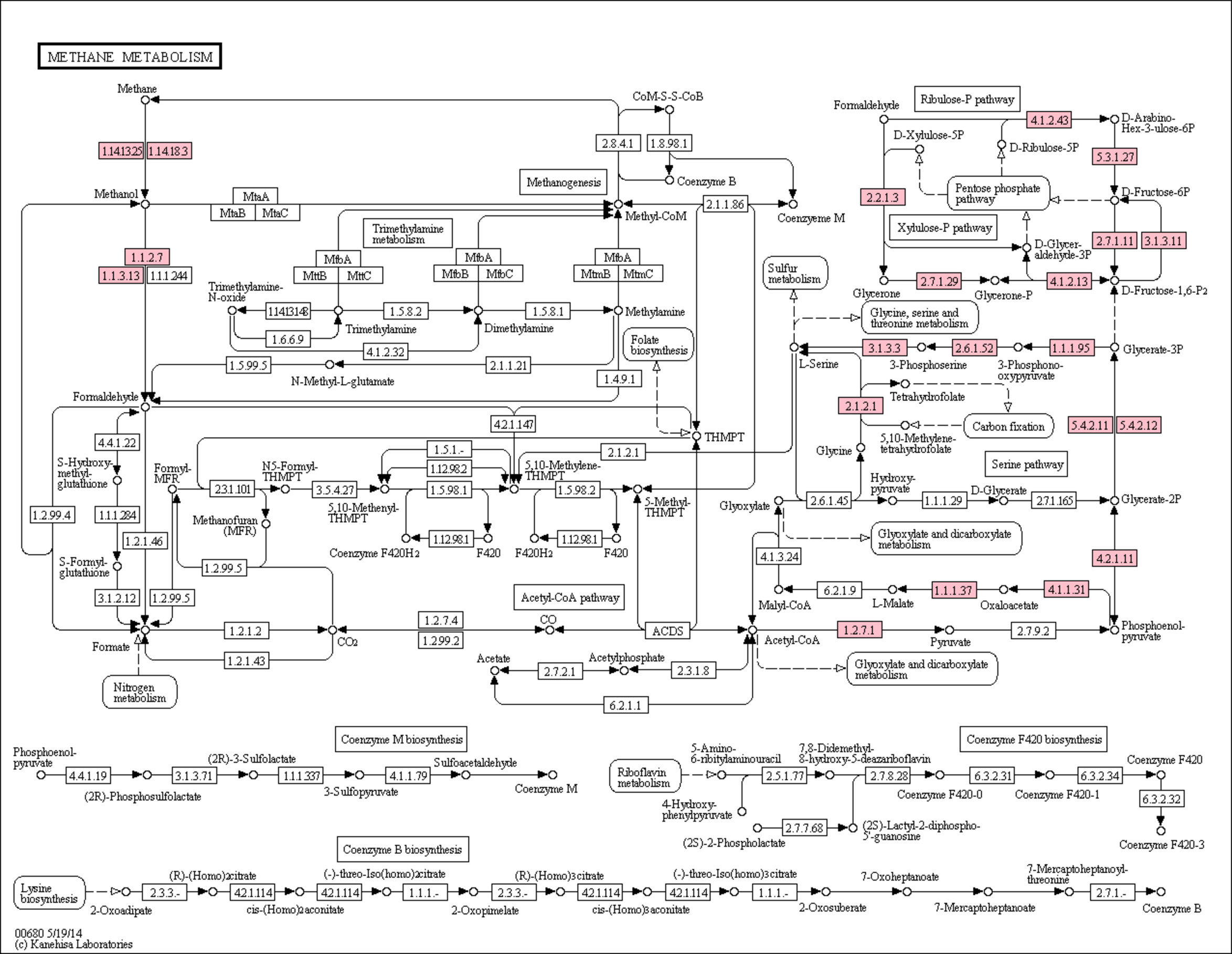

Supplement: S1 Fig — (TIF) [file pone.0128008.s001.tif]
