# Supplementary material for: Noteworthy Facts about a Methane-Producing Microbial Community Processing Acidic Effluent from Sugar Beet Molasses Fermentation
Source: PLoS One. 2015 May 22;10(5):e0128008. doi: 10.1371/journal.pone.0128008 (PMC4441513; doi:10.1371/journal.pone.0128008)
Supplement: S1 Table — (DOC) [file pone.0128008.s002.doc]

S1 Table. Number of reads assigned to respective taxonomic branches by MEGAN5.

File type: RMA2_FILE

Reads total: 516638

Assignments to nodes:

root: 516638

cellular organisms: 403816

Bacteria: 308946

Actinobacteria <phylum>: 6787

Actinobacteria: 6787

Actinobacteridae: 3371

Actinomycetales: 1265

Actinomycineae: 78

Actinomycetaceae: 78

Corynebacterineae: 183

Mycobacteriaceae: 60

Mycobacterium: 58

Micrococcineae: 218

Microbacteriaceae: 52

Propionibacterineae: 100

Nocardioidaceae: 52

Streptomycineae: 136

Streptomycetaceae: 136

Streptomyces: 133

Bifidobacteriales: 2003

Bifidobacteriaceae: 2003

Bifidobacterium: 1180

Bifidobacterium minimum: 105

Coriobacteridae: 3113

Coriobacteriales: 3113

Coriobacterineae: 3113

Coriobacteriaceae: 3113

Atopobium: 137

Collinsella: 245

Eggerthella: 231

Eggerthella sp. YY7918: 51

environmental samples <Eggerthella>: 50

Gordonibacter: 91

Gordonibacter pamelaeae: 91

Gordonibacter pamelaeae 7-10-1-b: 91

Olsenella: 137

Olsenella uli: 50

Slackia: 202

Slackia heliotrinireducens: 97

Slackia heliotrinireducens DSM 20476: 97

Aquificae <phylum>: 82

Aquificae: 82

Aquificales: 81

Armatimonadetes: 1084

Chthonomonadetes: 484

Chthonomonadales: 484

Chthonomonadaceae: 484

Chthonomonas: 484

Chthonomonas calidirosea: 484

Chthonomonas calidirosea T49: 484

Fimbriimonas: 214

Fimbriimonas ginsengisoli: 214

Fimbriimonas ginsengisoli Gsoil 348: 214

unclassified Armatimonadetes: 240

Armatimonadetes bacterium JGI 0000077-K19: 240

Bacteroidetes/Chlorobi group: 64735

Bacteroidetes: 64086

Bacteroidia: 50577

Bacteroidales: 50577

Bacteroidaceae: 16068

Bacteroides: 16068

Bacteroides caccae: 76

Bacteroides cellulosilyticus: 758

Bacteroides fragilis: 165

Bacteroides intestinalis: 61

Bacteroides oleiciplenus: 543

Bacteroides ovatus: 59

Bacteroides pyogenes: 171

Bacteroides pyogenes JCM 10003: 50

Bacteroides pyogenes JCM 6294: 61

Bacteroides sp. HPS0048: 159

Bacteroides uniformis: 80

environmental samples <Bacteroides>: 516

Bacteroides intestinalis CAG:315: 81

Marinilabiliaceae: 357

Anaerophaga: 119

Anaerophaga thermohalophila: 119

Porphyromonadaceae: 12993

Dysgonomonas: 688

Dysgonomonas gadei: 240

Dysgonomonas mossii: 207

Odoribacter: 111

environmental samples <Odoribacter>: 56

Paludibacter: 630

Paludibacter propionicigenes: 630

Paludibacter propionicigenes WB4: 630

Parabacteroides: 2146

environmental samples <Parabacteroides>: 72

Parabacteroides sp. CAG:409: 53

Parabacteroides distasonis: 185

Parabacteroides distasonis ATCC 8503: 150

Parabacteroides goldsteinii: 205

Parabacteroides merdae: 71

Porphyromonas: 175

Proteiniphilum: 6239

Proteiniphilum acetatigenes: 6239

Tannerella: 215

environmental samples <Tannerella>: 68

Tannerella sp. CAG:118: 63

Tannerella forsythia: 82

Tannerella forsythia ATCC 43037: 82

Prevotellaceae: 2573

Prevotella: 2381

environmental samples <Prevotella>: 240

Prevotella bergensis: 52

Prevotella dentalis: 63

Prevotella dentalis DSM 3688: 63

Prevotella multisaccharivorax: 74

Rikenellaceae: 715

Alistipes: 715

Alistipes indistinctus: 50

environmental samples <Alistipes>: 503

Alistipes sp. CAG:435: 54

Alistipes sp. CAG:831: 165

unclassified Bacteroidales: 321

unclassified Bacteroidales (miscellaneous): 296

Bacteroidales bacterium CF: 282

Cytophagia: 1034

Cytophagales: 1034

Cyclobacteriaceae: 236

Cytophagaceae: 508

Cytophaga: 96

Spirosoma: 52

Flammeovirgaceae: 160

Flavobacteriia: 1334

Flavobacteriales: 1291

Cryomorphaceae: 146

Fluviicola: 88

Fluviicola taffensis: 88

Fluviicola taffensis DSM 16823: 88

Owenweeksia: 50

Owenweeksia hongkongensis: 50

Owenweeksia hongkongensis DSM 17368: 50

Flavobacteriaceae: 1005

Flavobacterium: 267

Sphingobacteriia: 837

Sphingobacteriales: 836

Chitinophagaceae: 236

Niabella: 86

Niastella: 50

Niastella koreensis: 50

Niastella koreensis GR20-10: 50

Saprospiraceae: 120

Lewinella: 61

Sphingobacteriaceae: 398

Pedobacter: 143

Sphingobacterium: 83

unclassified Bacteroidetes: 494

unclassified Bacteroidetes (miscellaneous): 479

Bacteroidetes bacterium FH5: 384

Bacteroidetes oral taxon 274: 55

Chlorobi: 154

Chlorobia: 154

Chlorobiales: 154

Chlorobiaceae: 154

Chlorobium/Pelodictyon group: 91

Chlorobium: 69

Ignavibacteriae: 161

Ignavibacteria: 161

Ignavibacteriales: 161

Ignavibacteriaceae: 61

Ignavibacterium: 61

Ignavibacterium album: 61

Ignavibacterium album JCM 16511: 61

Melioribacteraceae: 75

Melioribacter: 75

Melioribacter roseus: 75

Melioribacter roseus P3M-2: 75

Chlamydiae/Verrucomicrobia group: 3880

Lentisphaerae: 79

Lentisphaeria: 79

Lentisphaerales: 79

Lentisphaeraceae: 79

Lentisphaera: 79

Lentisphaera araneosa: 79

Verrucomicrobia: 3722

Opitutae: 535

Opitutales: 242

Opitutaceae: 242

Opitutus: 92

Opitutus terrae: 92

Opitutus terrae PB90-1: 92

unclassified Opitutaceae: 141

Opitutaceae bacterium TAV5: 56

Puniceicoccales: 282

Puniceicoccaceae: 282

Coraliomargarita: 282

environmental samples <Coraliomargarita>: 258

Coraliomargarita sp. CAG:312: 258

Spartobacteria: 101

Chthoniobacter: 101

Chthoniobacter flavus: 101

unclassified Verrucomicrobia: 373

unclassified Verrucomicrobia (miscellaneous): 353

Verrucomicrobia bacterium SCGC AAA164-E04: 194

Verrucomicrobiae: 1225

Verrucomicrobiales: 1225

Verrucomicrobia subdivision 3: 1036

Pedosphaera: 1036

Pedosphaera parvula: 1036

Verrucomicrobiaceae: 129

Verrucomicrobium: 70

Verrucomicrobium spinosum: 64

Chloroflexi <phylum>: 1633

Anaerolineae: 557

Anaerolineales: 557

Anaerolineaceae: 557

Anaerolinea: 557

Anaerolinea thermophila: 557

Anaerolinea thermophila UNI-1: 557

Caldilineae: 88

Caldilineales: 88

Caldilineaceae: 88

Caldilinea: 88

Caldilinea aerophila: 88

Caldilinea aerophila DSM 14535 = NBRC 104270: 88

Chloroflexi: 128

Chloroflexales: 109

Chloroflexaceae: 91

Dehalococcoidia: 298

Dehalococcoidales: 195

Dehalococcoidaceae: 195

Dehalococcoides: 195

Dehalococcoides mccartyi: 195

Dehalococcoides mccartyi BTF08: 52

Ktedonobacteria: 80

Ktedonobacterales: 80

Ktedonobacteraceae: 80

Ktedonobacter: 80

Ktedonobacter racemifer: 80

Thermomicrobia: 51

unclassified Chloroflexi: 106

unclassified Chloroflexi (miscellaneous): 102

Chloroflexi bacterium oral taxon 439: 96

Cyanobacteria: 750

Nostocales: 88

Nostocaceae: 56

Oscillatoriophycideae: 383

Chroococcales: 129

Oscillatoriales: 195

Deferribacteres <phylum>: 116

Deferribacteres: 116

Deferribacterales: 116

Deferribacteraceae: 116

Denitrovibrio: 50

Denitrovibrio acetiphilus: 50

Denitrovibrio acetiphilus DSM 12809: 50

Deinococcus-Thermus: 120

Deinococci: 120

Deinococcales: 51

Elusimicrobia: 160

Elusimicrobia <class>: 140

Elusimicrobiales: 140

Elusimicrobiaceae: 140

Elusimicrobium: 140

Elusimicrobium minutum: 140

Elusimicrobium minutum Pei191: 140

environmental samples <Bacteria>: 961

uncultured bacterium: 900

Fibrobacteres/Acidobacteria group: 337

Acidobacteria: 226

Acidobacteriia: 90

Acidobacteriales: 90

Acidobacteriaceae: 90

unclassified Acidobacteriaceae: 55

Marinimicrobia: 89

Firmicutes: 73582

Bacilli: 3335

Bacillales: 1836

Bacillaceae: 532

Bacillus: 308

Paenibacillaceae: 737

Brevibacillus: 70

Paenibacillus: 565

Lactobacillales: 1306

Enterococcaceae: 473

Enterococcus: 441

Enterococcus faecalis: 209

Lactobacillaceae: 467

Lactobacillus: 451

Lactobacillus casei group: 87

Streptococcaceae: 151

Streptococcus: 115

Clostridia: 43983

Clostridiales: 41011

Clostridiaceae: 10505

Alkaliphilus: 82

Butyricicoccus: 186

Butyricicoccus pullicaecorum: 186

Clostridium: 9785

Clostridium asparagiforme: 138

Clostridium bolteae: 67

Clostridium botulinum: 76

Clostridium citroniae: 182

Clostridium hathewayi: 486

Clostridium phytofermentans: 61

Clostridium phytofermentans ISDg: 61

Clostridium saccharolyticum: 89

Clostridium saccharolyticum WM1: 82

Clostridium sp. 7_3_54FAA: 92

Clostridium sp. ATCC 29733: 60

Clostridium sp. D5: 147

Clostridium sp. MSTE9: 55

Clostridium symbiosum: 1035

Clostridium termitidis: 59

Clostridium ultunense: 65

environmental samples <Clostridium>: 2149

Clostridium sp. CAG:1024: 276

Clostridium sp. CAG:138: 60

Clostridium sp. CAG:226: 734

unclassified Clostridiaceae: 54

Youngiibacter: 157

Youngiibacter fragilis: 157

Clostridiales incertae sedis: 267

Clostridiales Family XI. Incertae Sedis: 102

Clostridiales Family XIII. Incertae Sedis: 78

Eubacteriaceae: 1854

Acetobacterium: 131

Acetobacterium woodii: 130

Acetobacterium woodii DSM 1030: 130

Eubacterium: 984

environmental samples <Eubacterium>: 261

Eubacterium sp. CAG:841: 70

Eubacterium acidaminophilum: 62

Eubacterium acidaminophilum DSM 3953: 62

Eubacterium limosum: 506

Eubacterium limosum KIST612: 506

Pseudoramibacter: 54

Pseudoramibacter alactolyticus: 54

Heliobacteriaceae: 86

Heliobacterium: 85

Heliobacterium modesticaldum: 85

Heliobacterium modesticaldum Ice1: 85

Lachnospiraceae: 1909

Blautia: 284

Blautia producta: 132

Butyrivibrio: 143

Dorea: 72

Marvinbryantia: 55

Marvinbryantia formatexigens: 55

Oribacterium: 73

Roseburia: 130

environmental samples <Roseburia>: 72

unclassified Lachnospiraceae: 716

Lachnospiraceae bacterium 3_1_57FAA_CT1: 374

Lachnospiraceae bacterium 7_1_58FAA: 66

Oscillospiraceae: 3519

Oscillibacter: 3519

environmental samples <Oscillibacter>: 187

Oscillibacter sp. CAG:155: 137

Oscillibacter sp. 1-3: 164

Oscillibacter valericigenes: 2202

Oscillibacter valericigenes Sjm18-20: 2202

Peptococcaceae: 2678

Dehalobacter: 394

Dehalobacter sp. E1: 132

Dehalobacter sp. FTH1: 92

Desulfitobacterium: 464

Desulfitobacterium dichloroeliminans: 70

Desulfitobacterium dichloroeliminans LMG P-21439: 70

Desulfitobacterium hafniense: 224

Desulfitobacterium hafniense Y51: 69

Desulfosporosinus: 326

Desulfosporosinus orientis: 86

Desulfosporosinus orientis DSM 765: 86

Desulfosporosinus youngiae: 91

Desulfotomaculum: 642

Desulfotomaculum acetoxidans: 130

Desulfotomaculum acetoxidans DSM 771: 130

Desulfotomaculum gibsoniae: 81

Desulfotomaculum gibsoniae DSM 7213: 81

Desulfotomaculum kuznetsovii: 52

Desulfotomaculum kuznetsovii DSM 6115: 52

Desulfotomaculum ruminis: 198

Desulfotomaculum ruminis DSM 2154: 198

Pelotomaculum: 86

Pelotomaculum thermopropionicum: 86

Pelotomaculum thermopropionicum SI: 86

Syntrophobotulus: 141

Syntrophobotulus glycolicus: 141

Syntrophobotulus glycolicus DSM 8271: 141

Thermincola: 62

Thermincola potens: 62

Thermincola potens JR: 62

Peptostreptococcaceae: 178

unclassified Peptostreptococcaceae: 152

[Clostridium] difficile: 61

Ruminococcaceae: 3538

Acetivibrio: 58

Acetivibrio cellulolyticus: 58

Anaerotruncus: 521

Anaerotruncus colihominis: 178

Anaerotruncus sp. G3(2012): 116

environmental samples <Anaerotruncus>: 60

Anaerotruncus sp. CAG:390: 56

Ethanoligenens: 168

Ethanoligenens harbinense: 168

Ethanoligenens harbinense YUAN-3: 168

Faecalibacterium: 532

environmental samples <Faecalibacterium>: 441

Faecalibacterium sp. CAG:1138: 50

Faecalibacterium sp. CAG:74: 383

Faecalibacterium prausnitzii: 74

Ruminococcus: 1181

environmental samples <Ruminococcus>: 965

Ruminococcus sp. CAG:382: 720

Subdoligranulum: 728

environmental samples <Subdoligranulum>: 303

Subdoligranulum sp. CAG:314: 303

Subdoligranulum sp. 4_3_54A2FAA: 331

Subdoligranulum variabile: 86

unclassified Ruminococcaceae: 168

[Clostridium] stercorarium: 70

Clostridium stercorarium subsp. stercorarium: 70

Clostridium stercorarium subsp. stercorarium DSM 8532: 70

Ruminococcaceae bacterium D16: 98

Syntrophomonadaceae: 4301

Syntrophomonas: 2747

Syntrophomonas wolfei: 2746

Syntrophomonas wolfei subsp. wolfei: 2746

Syntrophomonas wolfei subsp. wolfei str. Goettingen: 2746

Syntrophothermus: 466

Syntrophothermus lipocalidus: 466

Syntrophothermus lipocalidus DSM 12680: 466

unclassified Clostridiales: 644

Pseudoflavonifractor: 266

Pseudoflavonifractor capillosus: 266

unclassified Clostridiales (miscellaneous): 307

Clostridiales bacterium 1_7_47FAA: 176

Clostridiales bacterium NK3B98: 59

Halanaerobiales: 125

Halanaerobiaceae: 72

Thermoanaerobacterales: 758

Thermoanaerobacteraceae: 428

Moorella group: 66

Tepidanaerobacter: 74

Tepidanaerobacter acetatoxydans: 74

Tepidanaerobacter acetatoxydans Re1: 74

Thermacetogenium: 153

Thermacetogenium phaeum: 153

Thermacetogenium phaeum DSM 12270: 153

Thermoanaerobacterales Family III. Incertae Sedis: 87

Thermoanaerobacterales Family IV. Incertae Sedis: 144

Mahella: 144

Mahella australiensis: 144

Mahella australiensis 50-1 BON: 144

environmental samples <clostridial firmicutes>: 1549

Firmicutes bacterium CAG:103: 92

Firmicutes bacterium CAG:110: 57

Firmicutes bacterium CAG:114: 61

Firmicutes bacterium CAG:124: 85

Firmicutes bacterium CAG:129: 54

Firmicutes bacterium CAG:145: 50

Firmicutes bacterium CAG:170: 72

Firmicutes bacterium CAG:240: 51

Firmicutes bacterium CAG:272: 98

Erysipelotrichia: 689

Erysipelotrichales: 689

Erysipelotrichaceae: 689

Coprobacillus: 101

environmental samples <Coprobacillus>: 64

Holdemania: 75

Holdemania filiformis: 75

Solobacterium: 151

Solobacterium moorei: 151

unclassified Erysipelotrichaceae: 102

Negativicutes: 1241

Selenomonadales: 1241

Acidaminococcaceae: 329

Acidaminococcus: 108

Phascolarctobacterium: 176

Phascolarctobacterium sp. CAG:207: 102

Veillonellaceae: 842

Anaeromusa: 100

Anaeromusa acidaminophila: 100

Megasphaera: 94

Selenomonas: 86

Sporomusa: 77

Sporomusa ovata: 77

unclassified Firmicutes sensu stricto: 86

unclassified Firmicutes sensu stricto (miscellaneous): 86

Firmicutes bacterium ASF500: 77

Fusobacteria: 177

Fusobacteriia: 177

Fusobacteriales: 177

Fusobacteriaceae: 99

Fusobacterium: 80

Leptotrichiaceae: 76

Leptotrichia: 55

Nitrospirae: 82

Nitrospira <class>: 82

Nitrospirales: 82

Nitrospiraceae: 82

Planctomycetes: 1002

Planctomycetia: 966

Planctomycetales: 886

Planctomycetaceae: 869

Isosphaera: 54

Isosphaera pallida: 54

Isosphaera pallida ATCC 43644: 54

Planctomyces: 81

Planctomyces maris: 52

Rhodopirellula: 129

Schlesneria: 63

Schlesneria paludicola: 63

Singulisphaera: 64

Singulisphaera acidiphila: 64

Singulisphaera acidiphila DSM 18658: 64

unclassified Planctomycetaceae: 61

planctomycete KSU-1: 61

Proteobacteria: 27568

Alphaproteobacteria: 1411

Rhizobiales: 369

Bradyrhizobiaceae: 54

Phyllobacteriaceae: 65

Rhizobiaceae: 79

Rhodobacterales: 110

Rhodobacteraceae: 90

Rhodospirillales: 322

Acetobacteraceae: 206

Acetobacter: 82

Rhodospirillaceae: 106

Sphingomonadales: 177

Sphingomonadaceae: 163

unclassified Alphaproteobacteria: 59

Betaproteobacteria: 1251

Burkholderiales: 867

Alcaligenaceae: 366

Alcaligenes: 312

Alcaligenes faecalis: 168

Burkholderiaceae: 111

Burkholderia: 55

Comamonadaceae: 145

Oxalobacteraceae: 69

Sutterellaceae: 69

Sutterella: 59

Rhodocyclales: 116

Rhodocyclaceae: 116

delta/epsilon subdivisions: 17227

Deltaproteobacteria: 16930

Desulfarculales: 108

Desulfarculaceae: 108

Desulfarculus: 108

Desulfarculus baarsii: 108

Desulfarculus baarsii DSM 2075: 108

Desulfobacterales: 1460

Desulfobacteraceae: 1284

Candidatus Magnetoglobus: 108

Candidatus Magnetoglobus multicellularis: 108

Candidatus Magnetoglobus multicellularis str. Araruama: 108

Desulfatibacillum: 184

Desulfatibacillum alkenivorans: 184

Desulfatibacillum alkenivorans AK-01: 184

Desulfobacter: 59

Desulfobacterium: 304

Desulfobacterium autotrophicum: 100

Desulfobacterium autotrophicum HRM2: 100

environmental samples <Desulfobacterium>: 201

uncultured Desulfobacterium sp.: 201

Desulfococcus: 205

Desulfococcus multivorans: 59

Desulfococcus oleovorans: 142

Desulfococcus oleovorans Hxd3: 142

Desulfobulbaceae: 131

Desulfovibrionales: 6195

Desulfohalobiaceae: 59

Desulfomicrobiaceae: 62

Desulfomicrobium: 62

Desulfomicrobium baculatum: 62

Desulfomicrobium baculatum DSM 4028: 62

Desulfovibrionaceae: 5708

Bilophila: 66

Desulfovibrio: 5497

Desulfovibrio africanus: 51

Desulfovibrio desulfuricans: 260

Desulfovibrio desulfuricans subsp. desulfuricans: 131

Desulfovibrio desulfuricans subsp. desulfuricans str. ATCC 27774: 131

Desulfovibrio fructosivorans: 328

Desulfovibrio gigas: 51

Desulfovibrio gigas DSM 1382 = ATCC 19364: 51

Desulfovibrio magneticus: 305

Desulfovibrio magneticus RS-1: 251

Desulfovibrio sp. FW1012B: 65

Desulfovibrio sp. U5L: 92

Desulfovibrio sp. X2: 54

Desulfuromonadales: 1041

Geobacteraceae: 568

Geobacter: 510

Geobacter uraniireducens: 56

Geobacter uraniireducens Rf4: 56

Geopsychrobacter: 52

Geopsychrobacter electrodiphilus: 52

Pelobacteraceae: 267

Pelobacter: 267

Pelobacter carbinolicus: 60

Pelobacter carbinolicus DSM 2380: 60

Pelobacter propionicus: 190

Pelobacter propionicus DSM 2379: 190

environmental samples <delta subdivision>: 74

uncultured delta proteobacterium: 70

Myxococcales: 374

Cystobacterineae: 305

Myxococcaceae: 256

Corallococcus: 186

environmental samples <Corallococcus>: 183

Corallococcus sp. CAG:1435: 183

Syntrophobacterales: 4791

Syntrophaceae: 4523

Desulfobacca: 66

Desulfobacca acetoxidans: 66

Desulfobacca acetoxidans DSM 11109: 66

Desulfomonile: 157

Desulfomonile tiedjei: 157

Desulfomonile tiedjei DSM 6799: 157

Smithella: 838

Smithella sp. ME-1: 838

Syntrophus: 3132

Syntrophus aciditrophicus: 3132

Syntrophus aciditrophicus SB: 3132

Syntrophobacteraceae: 156

Syntrophobacter: 156

Syntrophobacter fumaroxidans: 156

Syntrophobacter fumaroxidans MPOB: 156

unclassified Deltaproteobacteria: 278

unclassified Deltaproteobacteria (miscellaneous): 202

delta proteobacterium NaphS2: 193

Epsilonproteobacteria: 267

Campylobacterales: 248

Campylobacteraceae: 194

Sulfurospirillum: 110

Gammaproteobacteria: 4594

Aeromonadales: 91

Succinivibrionaceae: 66

Succinimonas: 60

Succinimonas amylolytica: 60

Alteromonadales: 190

Alteromonadaceae: 78

Chromatiales: 342

Chromatiaceae: 141

Ectothiorhodospiraceae: 70

Halothiobacillaceae: 129

Halothiobacillus: 129

Halothiobacillus neapolitanus: 129

Halothiobacillus neapolitanus c2: 129

Enterobacteriales: 2393

Enterobacteriaceae: 2393

Enterobacter: 603

Enterobacter cloacae complex: 208

Escherichia: 54

Klebsiella: 314

Klebsiella oxytoca: 58

Methylococcales: 148

Methylococcaceae: 148

Oceanospirillales: 128

Halomonadaceae: 58

Halomonas: 58

Pseudomonadales: 405

Moraxellaceae: 247

Acinetobacter: 235

Pseudomonadaceae: 156

Pseudomonas: 145

Thiotrichales: 80

Thiotrichaceae: 53

unclassified Gammaproteobacteria: 98

Vibrionales: 77

Vibrionaceae: 73

Xanthomonadales: 71

Xanthomonadaceae: 61

Spirochaetes: 21398

Spirochaetia: 21398

Spirochaetales: 21398

Leptospiraceae: 129

Leptospira: 90

Spirochaetaceae: 21170

Sphaerochaeta: 15535

Sphaerochaeta coccoides: 953

Sphaerochaeta coccoides DSM 17374: 953

Sphaerochaeta globosa: 2960

Sphaerochaeta globosa str. Buddy: 2960

Sphaerochaeta pleomorpha: 2309

Sphaerochaeta pleomorpha str. Grapes: 2309

Spirochaeta: 424

Spirochaeta thermophila: 53

Treponema: 1366

Treponema azotonutricium: 86

Treponema azotonutricium ZAS-9: 86

Treponema brennaborense: 57

Treponema brennaborense DSM 12168: 57

Treponema bryantii: 58

Treponema caldaria: 105

Treponema caldaria DSM 7334: 105

Treponema primitia: 93

Treponema socranskii: 175

Synergistetes: 3247

Synergistia: 3127

Synergistales: 3127

Synergistaceae: 3127

Aminobacterium: 267

Aminobacterium colombiense: 267

Aminobacterium colombiense DSM 12261: 267

Dethiosulfovibrio: 66

Dethiosulfovibrio peptidovorans: 66

Jonquetella: 76

Pyramidobacter: 897

Pyramidobacter piscolens: 897

Synergistes: 744

Synergistes sp. 3_1_syn1: 744

Thermovirga: 59

Thermovirga lienii: 59

Thermovirga lienii DSM 17291: 59

Tenericutes: 70

Mollicutes: 70

Acholeplasmatales: 50

Acholeplasmataceae: 50

Acholeplasma: 50

Thermodesulfobacteria <phylum>: 64

Thermodesulfobacteria: 64

Thermodesulfobacteriales: 64

Thermodesulfobacteriaceae: 64

Thermotogae <phylum>: 274

Thermotogae: 274

Thermotogales: 274

Thermotogaceae: 274

Mesotoga: 102

unclassified Bacteria: 24949

Aminicenantes: 132

unclassified Aminicenantes: 132

Caldithrix: 86

Caldithrix abyssi: 86

candidate division EM 19: 70

unclassified candidate division EM 19: 70

candidate division KSB1: 287

unclassified candidate division KSB1: 287

Cloacimonetes bacterium SCGC AAA252-N05: 53

Cloacimonetes bacterium SCGC AAA252-O17: 105

candidate division NKB19: 203

unclassified candidate division NKB19: 203

Hydrogenedentes bacterium JGI 0000039-J10: 203

candidate division OP9: 57

unclassified candidate division OP9: 57

Cloacimonetes: 22742

Candidatus Cloacimonas: 3959

Candidatus Cloacimonas acidaminovorans: 3959

Candidatus Cloacimonas acidaminovorans str. Evry: 3959

unclassified Cloacimonetes: 821

Cloacimonetes bacterium JGI 0000039-A21: 142

Cloacimonetes bacterium JGI 0000039-G13: 112

Latescibacteria: 122

Candidatus Latescibacter: 71

Candidatus Latescibacter anaerobius: 71

unclassified Latescibacteria: 50

Poribacteria: 173

Poribacteria bacterium WGA-3G: 91

Archaea: 77818

Crenarchaeota: 103

Thermoprotei: 80

environmental samples <Archaea>: 89

Euryarchaeota: 76350

Archaeoglobi: 79

Archaeoglobales: 79

Archaeoglobaceae: 79

Archaeoglobus: 56

Halobacteria: 148

Halobacteriales: 148

Halobacteriaceae: 136

Methanobacteria: 190

Methanobacteriales: 190

Methanobacteriaceae: 161

Methanobacterium: 85

Methanococci: 55

Methanococcales: 55

Methanomicrobia: 53117

Methanocellales: 135

Methanocellaceae: 135

Methanocella: 135

Methanocella arvoryzae: 63

Methanocella arvoryzae MRE50: 63

Methanomicrobiales: 46087

Methanocorpusculaceae: 8379

Methanocorpusculum: 8379

Methanocorpusculum labreanum: 8379

Methanocorpusculum labreanum Z: 8379

Methanomicrobiaceae: 34558

Methanoculleus: 28788

environmental samples <Methanoculleus>: 254

Methanoculleus sp. CAG:1088: 254

Methanoculleus bourgensis: 3527

Methanoculleus bourgensis MS2: 3527

Methanoculleus marisnigri: 7410

Methanoculleus marisnigri JR1: 7410

Methanofollis: 3860

Methanofollis liminatans: 3859

Methanoplanus: 418

Methanoplanus limicola: 180

Methanoplanus petrolearius: 184

Methanoplanus petrolearius DSM 11571: 184

Methanoregulaceae: 517

Methanoregula: 203

Methanoregula formicica: 151

Methanoregula formicica SMSP: 151

Methanosphaerula: 249

Methanosphaerula palustris: 249

Methanosphaerula palustris E1-9c: 249

Methanospirillaceae: 137

Methanospirillum: 137

Methanospirillum hungatei: 137

Methanospirillum hungatei JF-1: 137

Methanosarcinales: 2713

Methanosaetaceae: 1896

Methanosaeta: 1896

Methanosaeta concilii: 1733

Methanosaeta concilii GP6: 1733

Methanosaeta harundinacea: 72

Methanosaeta harundinacea 6Ac: 72

Methanosarcinaceae: 743

Methanosarcina: 457

Methanosarcina acetivorans: 91

Methanosarcina acetivorans C2A: 91

Methanosarcina barkeri: 83

Methanosarcina barkeri str. Fusaro: 83

Methanosarcina mazei: 88

unclassified Methanomicrobia: 3063

Methanomassiliicoccus: 3063

Candidatus Methanomassiliicoccus intestinalis: 878

Candidatus Methanomassiliicoccus intestinalis Issoire-Mx1: 878

Methanomassiliicoccus luminyensis: 1117

Thermococci: 146

Thermococcales: 146

Thermococcaceae: 146

Pyrococcus: 61

Pyrococcus horikoshii: 55

Pyrococcus horikoshii OT3: 55

Thermococcus: 62

Thermoplasmata: 1640

Thermoplasmatales: 1638

unclassified Thermoplasmatales: 1557

Thermoplasmatales archaeon BRNA1: 1508

unclassified Euryarchaeota: 1783

Aciduliprofundum: 66

Candidatus Methanomethylophilus: 1694

Candidatus Methanomethylophilus alvus: 1694

Candidatus Methanomethylophilus alvus Mx1201: 1694

Eukaryota: 355

Opisthokonta: 173

Fungi: 54

Metazoa: 89

Eumetazoa: 85

Bilateria: 83

Viruses: 91

dsDNA viruses, no RNA stage: 82

Caudovirales: 67

unclassified sequences: 97

environmental samples <unclassified>: 97

prokaryotic environmental samples: 56

uncultured prokaryote: 51

Not assigned: 30035

Low complexity: 80062
